# Therapeutic Hints

**Published:** 1895-04

**Authors:** C. M. Boger

**Affiliations:** Parkersburg, W. Va.


### THERAPEUTIC HINTS.

C. M. Boger, M. D., Parkersburg, W. Va.

### Cyclamen.

When opposite mental states alternate think of Cyclamen as well as Ignatia. Cyclamen symptoms are very similar to those of Pulsatilla, but with opposite modalities, its menstrual flow is more profuse while at rest; in this respect being also just the reverse of Pulsatilla. This symptom is decisive, and led me to make a complete cure in a case of amenorrhœa, whose previous history showed this symptom.

### Sepia.

Supra-orbital pain, alternating from side to side, does not always mean Lac-canin., but often indicate *Sepia*, and has been verified.

### Graphites.

The Graphites patient is an overgrown Pulsatilla subject, with a well-developed motive temperament, large bones, and high forehead, slower in movement, but just as mild as Pulsatilla; apt to become obese and suffer from constipation and skin symptoms, especially chaps and fissures. Pusatilla has aggravation from milk, while Graphites has amelioration from warm milk. This is an important and practical distinction.

Periodicity of seven days, Canth., Croc., Gels, Nux-mos., Phos., *Phgt.*, Sang., Sep., Sil., Sulph.

